# Adipocytes Provide Fatty Acids to Acute Lymphoblastic Leukemia Cells

**DOI:** 10.3389/fonc.2021.665763

**Published:** 2021-04-22

**Authors:** Jonathan Tucci, Ting Chen, Katherine Margulis, Etan Orgel, Rebecca L. Paszkiewicz, Michael D. Cohen, Matthew J. Oberley, Rachel Wahhab, Anthony E. Jones, Ajit S. Divakaruni, Cheng-Chih Hsu, Sarah E. Noll, Xia Sheng, Richard N. Zare, Steven D. Mittelman

**Affiliations:** ^1^ Diabetes and Obesity Program, Center for Endocrinology, Diabetes and Metabolism, Children’s Hospital Los Angeles, Los Angeles, CA, United States; ^2^ Division of Pediatric Endocrinology, University of California Los Angeles (UCLA) Children’s Discovery and Innovation Institute, David Geffen School of Medicine UCLA, Los Angeles, CA, United States; ^3^ Department of Chemistry, Stanford University, Stanford, CA, United States; ^4^ The Institute for Drug Research, School of Pharmacy, The Hebrew University of Jerusalem, Jerusalem, Israel; ^5^ Cancer and Blood Disease Institute, Children’s Hospital Los Angeles, Los Angeles, CA, United States; ^6^ Department of Pathology, Children’s Hospital Los Angeles, Los Angeles, CA, United States; ^7^ Department of Molecular and Medical Pharmacology, UCLA David Geffen School of Medicine, Los Angeles, CA, United States; ^8^ Department of Chemistry, National Taiwan University, Taipei, Taiwan

**Keywords:** adipocytes, FFA, microenvironment, leukemia, lipid droplets

## Abstract

**Background:**

There is increasing evidence that adipocytes play an active role in the cancer microenvironment. We have previously reported that adipocytes interact with acute lymphoblastic leukemia (ALL) cells, contributing to chemotherapy resistance and treatment failure. In the present study, we investigated whether part of this resistance is due to adipocyte provision of lipids to ALL cells.

**Methods:**

We cultured 3T3-L1 adipocytes, and tested whether ALL cells or ALL-released cytokines induced FFA release. We investigated whether ALL cells took up these FFA, and using fluorescent tagged BODIPY-FFA and lipidomics, evaluated which lipid moieties were being transferred from adipocytes to ALL. We evaluated the effects of adipocyte-derived lipids on ALL cell metabolism using a Seahorse XF analyzer and expression of enzymes important for lipid metabolism, and tested whether these lipids could protect ALL cells from chemotherapy. Finally, we evaluated a panel of lipid synthesis and metabolism inhibitors to determine which were affected by the presence of adipocytes.

**Results:**

Adipocytes release free fatty acids (FFA) when in the presence of ALL cells. These FFA are taken up by the ALL cells and incorporated into triglycerides and phospholipids. Some of these lipids are stored in lipid droplets, which can be utilized in states of fuel deprivation. Adipocytes preferentially release monounsaturated FFA, and this can be attenuated by inhibiting the desaturating enzyme steroyl-CoA decarboxylase-1 (SCD1). Adipocyte-derived FFA can relieve ALL cell endogenous lipogenesis and reverse the cytotoxicity of pharmacological acetyl-CoA carboxylase (ACC) inhibition. Further, adipocytes alter ALL cell metabolism, shifting them from glucose to FFA oxidation. Interestingly, the unsaturated fatty acid, oleic acid, protects ALL cells from modest concentrations of chemotherapy, such as those that might be present in the ALL microenvironment. In addition, targeting lipid synthesis and metabolism can potentially reverse adipocyte protection of ALL cells.

**Conclusion:**

These findings uncover a previously unidentified interaction between ALL cells and adipocytes, leading to transfer of FFA for use as a metabolic fuel and macromolecule building block. This interaction may contribute to ALL resistance to chemotherapy, and could potentially be targeted to improve ALL treatment outcome.

## Introduction

Obesity increases the incidence and mortality of many cancers (1), including leukemia. Leukemia is the most common cancer in children and one of the top ten in adults (2). In children with acute lymphoblastic leukemia (ALL), obesity increases relapse rate by ~50% (3, 4) and risk of detectible minimal residual disease after the first month of chemotherapy more than two-fold (5). While many genetic, physiological, behavioral, and environmental factors could contribute to these effects, we and others have found that adipocytes protect cancer cells (6–13).

One way adipocytes support cancer cells is through provision of lipids (9–11, 14, 15). Adipocytes provide long-term storage of lipids, which are released in times of fasting or stress. However, local signals from cancer cells can induce lipolysis and free fatty acid release. These fatty acids are then available to be used by cancer cells; however, whether this occurs in ALL is unknown. Further, which lipids are provided to nearby cancer cells and how these affect metabolism and survival is not fully understood, particularly in ALL. These are important questions, as strategies are being developed to target lipid biosynthesis or metabolism in several cancers (16–24). Because the bone marrow is an adipocyte-rich environment, these interactions could be particularly relevant to hematologic cancers. Therefore, in the present study we evaluated whether ALL cells induce adipocyte lipolysis and utilize adipocyte-derived free-fatty acids (FFA).

## Materials and Methods

### Materials

Media and supplements were supplied by Gibco (Waltham, MA) unless stated otherwise. Corning^®^ Matrigel^®^ was from Corning (Corning, NY). Isoproterenol, daunorubicin, vincristine, etomoxir, T863, and CP-640186 were obtained from MedChemExpress (Monmouth Junction, NJ). Recombinant human TNFα was obtained from Biolegend (San Diego, CA), recombinant human CCL2, CCL17, IL-16, CCL5, and PDGF-AA and TNFα antibody and CCL5 antibody were obtained from R&D Systems (Minneapolis, MN). TOFA (5-(tetradecyloxy)-2-furoic acid), PluriSln1, Cay10566, ±-C75, a922500, cvt-12012, gassofermate, orlistat, cerulenin, pf-06424439, SSO (sulfosuccinimidyl oleate sodium salt), and fenofibrate were obtained from Cayman chemical (Ann Arbor, Michigan). Fasnall was obtained from Sigma-Aldrich (St. Louis, MO). SCD1 inhibitor was obtained from BioVision (Milpitas, CA). St1326 was obtained from Avanti Polar Lipids (Alabaster, Alabama). Infliximab was obtained from the Children’s Hospital Los Angeles pharmacy. PDGF-AA antibody and IL-16 antibody were obtained from EMD Millipore (Burlington, MA). Sodium palmitate and sodium oleate (Sigma-Aldrich) were conjugated to FFA-free bovine serum albumin (Thermo Fisher Scientific, San Jose, CA) at 37°C for 1 hour to create 1 mM palmitic or oleic acid stock solutions. BODIPY^®^ 500/510 C1, C12 (4,4-Difluoro-5-Methyl-4-Bora-3a,4a-Diaza-s-Indacene-3-Dodecanoic Acid), a fluorescently tagged free-fatty acid, was obtained from Molecular Probes (Waltham, MA).

### Human Samples

Plasma, ALL cells, and bone marrow smears and biopsy specimens were obtained from patients 10-19 years old at diagnosis with NCI/Rome high-risk B-ALL and after induction chemotherapy, ~day #29. This study was approved by the CHLA IRB with written documentation of assent/consent prior to collection of samples (NCT#02708108).

Anonymized remnant human adipose tissue was obtained through the UCLA Department of Pathology and Laboratory Medicine’s Translational Pathology Core Laboratory, approved by the UCLA IRB. Adipose tissue samples were kept in PBS on ice for up to 1 hour, cut into small pieces (~100 mg), and incubated in RPMI with 10% FBS overnight. Individual pieces were then put in fresh media, which was collected 24 hours later for measurement of FFA and glycerol concentrations. In some experiments, adipocytes and the stromovascular fractions (SVF) were isolated. Briefly, adipose tissue was minced in liberase solution (0.035 mg/mL DMEM/F12 per 0.5 gram fat), placed in the tissue culture incubator with the cap loosened for 15 minutes, incubated 30-60 minutes with a stir bar in a 37°C water bath, filtered through a 300 µm strainer, and allowed to sit until a clear layer of adipocytes floated on the top. The infranatant was removed and centrifuged at 500*g* for 10 minutes at room temperature, and SVF cells resuspended and subjected to RBC lysis. SVF cells were differentiated into adipocytes as previously described (25). The adipocyte layer was washed 2-3 times with media, transferred to DMEM/F12 with 2% BSA, 1:1000 insulin and dexamethasone in a 1:10 volume ratio of cells:media (26).

### Mouse Samples

Mouse use was approved by the CHLA IACUC and performed in accordance with the USPHS Policy on Humane Care and Use of Laboratory Animals. 4-6 week old male C57BL/6J mice were anesthetized with ketamine and xylazine and perfused with heparinized saline until liver clearing. Femurs were removed and bone marrow extruded.

### Tissue Culture

Murine pre-B 8093 ALL cells have been previously described (27). Human ALL cell lines SupB15, Molt4, and RS4;11, murine 3T3-L1 pre-adipocytes, and murine OP9 cells were from ATCC (Manassas, VA). Human ALL cell lines BV173 SD-1, SEM and Nalm-6 were from DSMZ (Braunschweig, Germany). All human cell lines were authenticated by short tandem repeats by the University of Arizona Genetics core on 11/2016, and tested negative for mycoplasma.

Murine and human ALL cells were cultured as previously described (28), with IL-3 (0.66 nM; Peprotech) and β-mercaptoethanol (55 µM) added to 8093 cells during every passage. 3T3-L1 cells were cultured and differentiated as previously described (29). All cells were cultured in incubators at 37°C with 5% CO_2_ and counts determined by trypan blue exclusion manually or by Countess II FL Automated Cell Counter (Thermo Fisher).

Non-leukemic precursor pre-B cells were obtained after extrusion of murine bone marrow into ACK lysis buffer (BD Biosciences, San Jose, CA), followed by 15 minute incubation at room temperature. After red blood cell lysis, cells were centrifuged and washed in cell staining buffer. Pre-B cells were isolated by FACS, described below, and then directly cultured on an OP-9 feeder layer in OPTI-MEM media with 10% FBS, 10 μg/mL gentamicin, 0.66 nM IL-7 (Peprotech, Rocky Hill, NJ) and 55 µM β-mercaptoethanol. Cells were passaged onto new OP9 feeder layers every 2-3 days and used within 3 weeks.

ALL cells were co-cultured in transwells over adipocytes, fibroblasts, or no cells as previously described (12). Conditioned media were prepared by culturing various cells or cell combinations in complete media for 48 hours.

### Adipocyte Lipid Tracing

To label adipocytes with BODIPY, differentiated 3T3-L1 adipocytes were serum-starved for 3 hours, then treated with 10 μM BODIPY and 100 nM insulin for 1 hour. Adipocytes were then washed 5 times with pre-warmed complete media and co-cultured under ALL cells in transwells. To label adipocytes with ^13^C lipids, 3T3-L1 pre-adipocytes were differentiated as above, but using glucose-free DMEM base media supplemented with 12.5 mM U-^13^C-glucose (Sigma) and 12.5 mM normal glucose for the first 7 days of differentiation. BV173 cells were plated on coverslips in transwells and suspended above ^13^C labeled adipocytes for various timepoints. After co-culture, coverslips were washed twice with cold PBS and dried onto slides or wells of plates, cell-side up. Coverslips were stored at -80°C until analysis.

### Microscopy

Adipocytes, ALL cells and human bone marrow smears were fixed in 4% paraformaldehyde and stained with Oil Red O and DAPI (Sigma) following the manufacturer’s protocol. Upon staining, samples were viewed under a Zeiss Axio Observer microscope and pictures taken at 40x. For lipid quantification, a blinded observer took pictures and quantified red pixel count normalized to number of adipocyte nuclei in 10 random fields, using ImageJ (NIH).

For confocal microscopy, ALL cells were grown in transwells on poly-D-lysine coated coverslips to promote adherence. Transwells were then placed over adipocytes pre-labeled with BODIPY-FFA for 72 hours. Images were acquired with an LSM 700 confocal system mounted on an AxioObserver.Z1 microscope equipped with a 63x/1.4 Plan-APOCHROMAT objective lens and controlled with ZEN 2009 software (Carl Zeiss Microscopy, Thornwood, NY). A 488 nm laser and 560 nm long-pass filter were used for fluorescence excitation and emission.

### Flow Cytometry

For determination of BODIPY uptake by ALL cells, cells were harvested from Transwell cultures, washed 3 times with cold PBS, resuspended in cell staining buffer and stained with 1 μg/mL DAPI. Cells were analyzed using flow cytometry on the LSR II Analyzer (BD Biosciences, San Jose, CA). BODIPY-FFA (excitation/emission is 500/510) were detected using the GFP channel. The median fluorescence of BODIPY-FFA were determined from histograms for each sample.

For isolation of murine pre-B cells, marrow cells were labeled with FITC-conjugated anti-mouse CD127, APC-conjugated anti-mouse CD19 (both from Biolegend, San Diego, CA) and DAPI, following manufacturer’s protocol. Cells positive for both CD19 and CD127 were isolated on a BD FACSAria-I cell sorter.

### Assays

FFA and glycerol concentrations were measured using colorimetric kits (NEFA-HR (2), Wako Pure Chemicals Industries, Ltd., Osaka, Japan, and MAK117-1KT, Sigma) following manufacturer’s protocols. Cytokines were measured by Eve Technology Corporation (Calgary, Canada) Human Cytokine 65-Discovery Assay.

### Lipidomic Analysis

Nanospray desorption electrospray ionization mass spectrometry (nanoDESI-MS) was performed in the Zare Laboratory at Stanford. Briefly, two fused silica capillary tubes (350/250 μm O.D./I.D.) were placed in close proximity above the sample surface. A high voltage was applied to the syringe needle, and solvent was continuously perfused, such that a liquid microjunction formed between the capillaries at the sample surface. Desorbed lipids were then aspirated through the second capillary toward the mass spectrometer inlet, where electrospray ionization and subsequent detection occurred (30–32). NanoDESI-MS was performed in a negative ion mode at *m/z* 200–1,200, with methanol:acetonitrile:ethyl acetate 50:35:15 (vol/vol/vol) as the solvent at 2.5 µL/min flow rate. Spray voltage was set to 2.4 kV, and electrospray ions generated by nanoDESI were introduced to an LTQ-Orbitrap-XL mass spectrometer (Thermo Fisher). MS resolving power of 100,000 was chosen in order to discern ^13^C-labeled fatty acid peaks from peaks of other lipid species. Because glucose carbons generally enter FFA *via* two carbon units through acetyl-CoA, ^13^C_2_ enrichment of a FFA was quantified as the peak area of M+2.007 divided by the peak area of the unlabeled FFA, M, of the same spectrum. This ratio was then normalized by the natural abundance of the ^13^C_2_-isotope peak, calculated from the carbon isotopic distribution pattern, and multiplied by 100 to obtain a percentage.

### Gene Expression

ALL cells were washed, resuspended in RNAProtect (Qiagen, Valencia, CA) and RNA extracted with RNEasy Mini kits (Qiagen). RNA concentration and quality were determined by NanoDrop (Thermo Fisher) and then converted to cDNA using the High Capacity 1st Strand Synthesis kit (Applied Biosystems). Expressions of fatty acid synthase (*FASN*), acetyl-CoA carboxylase (*ACC1*), stearoyl-CoA desaturase 1 (*SCD1*) and carnitine O-palmitoyl transferase 1A (*CPT1A*) were quantified by rtPCR on the ABI7900HT (Applied Biosystems, Waltham, MA) using Power SYBR Green PCR Master Mix (Applied Biosystems). Primers were derived from (33). Transcript levels were normalized to β-actin (Forward: 5’-ACAGAGCCTCGCCTTTGCCG-3’; Reverse: 5’-CGATGCCGTGCTCGATGGGG-3’).

### Western Blots

Cells were washed in cold PBS and resuspended in RIPA lysis buffer containing 0.1% Tween-20 and supplemented with phenylmethylsulfonyl fluoride, protease inhibitor cocktail, and phosphatase inhibitor cocktail. Samples were homogenized using a microcentrifuge tube pestle, and lysates snap frozen and stored at -80° until use. Protein concentrations were quantified by bicinchoninic acid assay (Pierce Biotechnology).

Whole cell lysates were run on 4-12% SDS-PAGE gel and blotted onto polyvinylidene difluoride membranes, blocked using Intercept blocking buffer (Li-Cor), and probed with mouse anti-human CPT1A (Abcam), rabbit anti-human phospho-PDH (Cell Signaling Technology), or rabbit anti-human PDH antibodies (Cell Signaling Technology). Rabbit anti-human GAPDH and mouse anti-human alpha tubulin were included as loading controls for CPT1a and PDH/pPDH respectively (Cell Signaling Technology). Blots were then probed with anti-rabbit DyLight 800 and anti-mouse DyLight 488 secondary antibodies (Invitrogen) followed by multiplex fluorescent imaging using Bio-Rad Chemidoc Touch Imaging System (Bio -Rad, Hercules CA). Densitometric analysis of bands was performed with ImageJ. CPT1A expression was normalized to GAPDH. Phosphorylated PDH and PDH were normalized to alpha tubulin.

### Respirometry and Lactate Efflux

Determining the standard bioenergetic profile of ALL cells cultured alone or with adipocytes: BV173 cells were cultured in transwells alone or over differentiated 3T3-L1 adipocytes, after which they were spun onto Seahorse XF96 plates (600*g* for 5 min) coated with Cell-Tak (Corning C354240, according to manufacturer’s instructions) at 3x10^5^ cells/well. Oligomycin (2 μM), FCCP (two sequential pulses of 300 nM), and rotenone (200 nM) with antimycin A (1 μM) were added acutely to the wells, and respiratory parameters calculated as in (34). Cells were assayed in standard conditions of DMEM media (Sigma) supplemented with 5 mM HEPES, 8 mM glucose, 2 mM glutamine, and 2 mM pyruvate. Lactate efflux was measured by correcting rates of extracellular acidification for (i) the scaling factor of the microplate sensor coverage and (ii) confounding respiratory acidification; rates of cellular ATP production were also calculated using these parameters, as described in (35).

Determining the effects of adipocytes on ALL cell oxidation of long-chain fatty acids: Oleate-driven respiration was quantified in DMEM supplemented with 5 mM HEPES, 0.5 mM glucose, and 0.5 mM carnitine. Oleic acid (O1008) was conjugated to BSA by adding it to a warmed solution of 10% (w/v) fatty acid-free BSA at a molar ratio of 6:1. The pH was adjusted to 7.2 with NaOH, cooled, and sterile-filtered, as was a matched BSA control. BSA or oleate-conjugated BSA was added to the assay medium at 0.5% (w/v), corresponding to a free oleate of ~700 nM (36). The “oleate effect” was defined as the difference in basal respiration between cells given oleate-conjugated BSA or control BSA for each condition. Respiration rates were corrected for background/non-mitochondrial respiration with rotenone and antimycin A as above.

Determining mitochondrial long-chain fatty acid oxidation rates: Palmitoyl CoA-driven respiration was quantified in plasma membrane-permeabilized BV173 cells. Permeabilized cells were given palmitoyl-CoA and carnitine, as described in (37), in MAS medium supplemented with 0.2% (w/v) fatty acid-free BSA, 3 nM recombinant perfringolysin O, 4 mM ADP, 40 μM palmitoyl CoA, 0.5 mM carnitine, and 0.5 mM malate. Respiration rates were corrected for background/non-mitochondrial respiration as above.

### Statistical Analysis

All statistical tests were performed with GraphPad Prism (GraphPad Software, Inc., La Jolla, CA) or Microsoft Excel. EC_50_ was calculated for each experiment by fitting individual dose response curves using least squares to log(drug) vs. normalized cell count on GraphPad Prism. Paired and unpaired two-sided student’s *t*-tests were used to compare groups. Log transformation was used for non-normally distributed data. Data are presented as mean ± SD, with significance considered p<0.05.

## Results

### ALL Cells Stimulate Adipocyte Lipolysis

**Figure 1:**
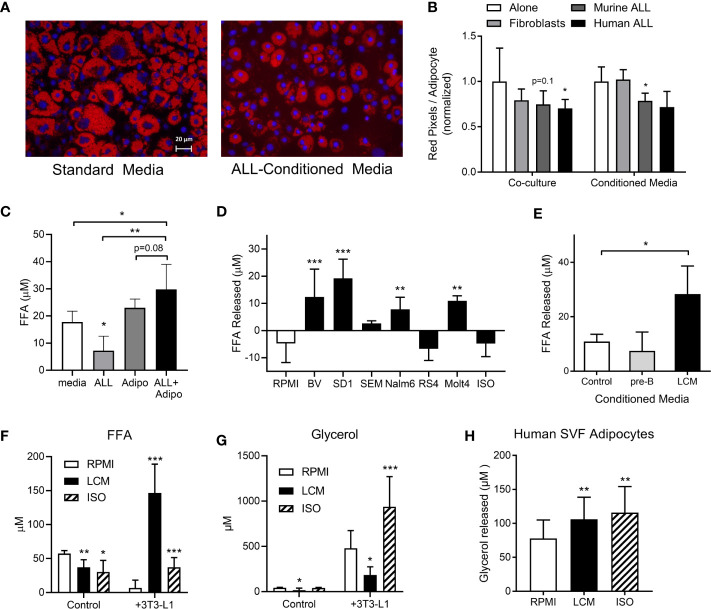
3T3-L1 adipocytes release FFA in the presence of ALL cells. **(A)** Representative images of adipocytes after 72-hour incubation in control (left) or 8093 conditioned media (LCM; right; Red = Oil Red O; Blue = DAPI counterstain). **(B)** Adipocyte lipid content after 72-hour co-culture (left) or culture in conditioned media (right) of 3T3-L1 fibroblasts, murine (8093) ALL cells, or human (BV173) ALL cells. Lipid content was quantified by pixel count of Oil Red O stained images divided by number of adipocytes in each image. Values are normalized to control conditions (20 images per condition per n; n=3) **(C)** FFA concentration in media after BV173 ALL and 3T3-L1 adipocytes were cultured for 24 hours alone or together in transwell systems (n=6). **(D)** FFA release from 3T3-L1 adipocytes after 24 hours in standard media or LCM from various ALL cell lines. ISO = 1 mM isoproterenol. n= 5. *’s represent significance vs. RPMI alone. **(E)** FFA released by adipocytes into media after 24 hour incubation with complete media, non-leukemic pre-B cell conditioned media or BV173 LCM (n=3). **(F)** FFA concentrations in RPMI, BV173 LCM, or 1 mM isoproterenol culture alone (left) or over adipocytes (right). *represent significance vs. RPMI bars (n=6-7). **(G)** Glycerol concentrations from the same experiments described in **(F)**. **(H)** Glycerol released from human SVF-derived adipocytes after culture with RPMI, LCM, or 1 mM isoproterenol (n=6). *p < 0.05, **p < 0.01, ***p < 0.001.

During routine co-cultures, we observed that adipocytes cultured under ALL-containing transwells had less lipid than those cultured alone. Lipid staining confirmed that adipocytes cultured directly with murine or human ALL, or in their conditioned media (LCM), had less lipid, though this did not reach significance for all conditions (**Figures 1A, B**). 3T3-L1 adipocytes had no net effect on media FFA concentration, while adipocytes co-culture with BV173 human ALL cells resulted in a net FFA increase, particularly compared to ALL cells cultured alone (**Figure 1C**). Media conditioned by some human ALL cell lines stimulated 3T3-L1 adipocyte FFA release (**Figure 1D**), while media conditioned by non-leukemic murine pre-B cells did not (**Figure 1E**). Interestingly, while LCM stimulated significant 3T3-L1 FFA release (**Figure 1F**), adipocytes released less glycerol when cultured in LCM (**Figure 1G**). These data imply that LCM did not increase lipolysis rate, but rather reduced adipocyte FFA reuptake and/or re-esterification. In contrast, isoproterenol greatly increased glycerol release, but not net FFA release.

We next tested whether ALL cells would stimulate FFA release from human adipocytes. Because human adipocyte culture models are less robust than murine, we tested multiple. Human adipocytes differentiated from fresh adipose stromal vascular fraction (SVF) showed net FFA uptake under basal conditions, which was not different in LCM (not shown). However, LCM and isoproterenol both stimulated modest amounts of glycerol release from these human adipocytes (**Figure 1H**). The other models we tested, including whole adipose tissue explants and isolated primary adipocytes cultured in matrigel, showed no significant effect of LCM on either FFA or glycerol release (not shown). Thus, ALL induces a robust release of FFA in 3T3-L1 adipocytes, while human models show less consistent effects.

### ALL Cytokines May Not Drive Adipocyte FFA Release

**Figure 2:**
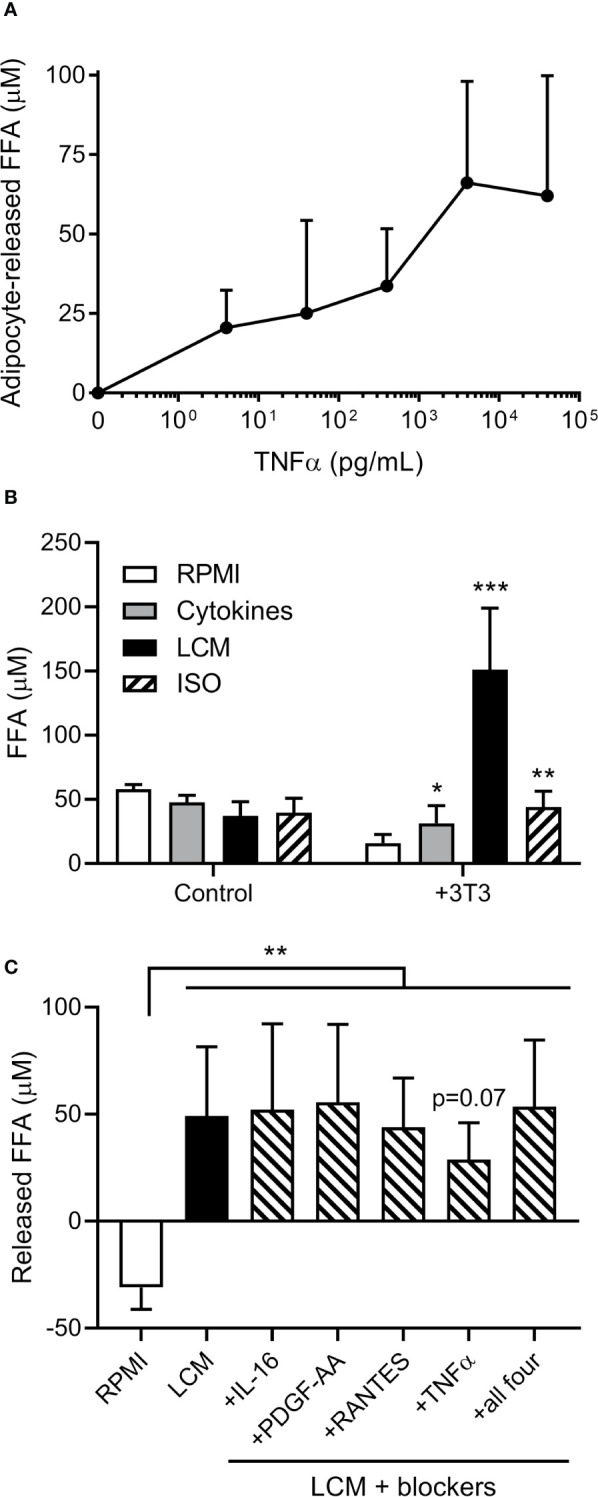
Effects of cytokines on 3T3-L1 adipocyte FFA release. **(A)** Measurement of adipocyte FFA release following TNFα exposure for 24 hours at various doses. (n=3/dose). **(B)** FFA concentrations in various media incubated alone or over 3T3-L1 adipocytes (n=6); *, **, *** indicate significance compared to RPMI alone over 3T3. **(C)** Net release of FFA from 3T3-L1 adipocytes after 24 hour incubation with LCM in the presence of four different cytokine receptor blockers. Blockers were added to LCM 30 minutes prior to adipocytes. (n=5) *p<0.05, **p<0.01, ***p<0.001.

As cytokines are known to induce adipocyte lipolysis, we investigated which are released by human ALL cells. A 65 human cytokine array revealed 21 cytokines were significantly or borderline higher in BV173 LCM than standard media with FBS (Table S1). TNFα induced FFA release in a dose-dependent manner (**Figure 2A**). We selected six cytokines with known effects on lipolysis [TNFα, RANTES (CCL5), CCL17, MCP-1 (CCL2), IL-16, PDGF-AA], and cultured adipocytes in their approximate measured concentrations. All six cytokines together induced modest adipocyte FFA release, but substantially less than LCM (**Figure 2B**). Monoclonal blocking antibodies to four of the cytokines did not attenuate this effect, though Infliximab, a monoclonal antibody to TNFα, tended to reduce LCM-stimulated FFA release (**Figure 2C**).

### ALL Cells Take Up Adipocyte-Derived FFA

**Figure 3:**
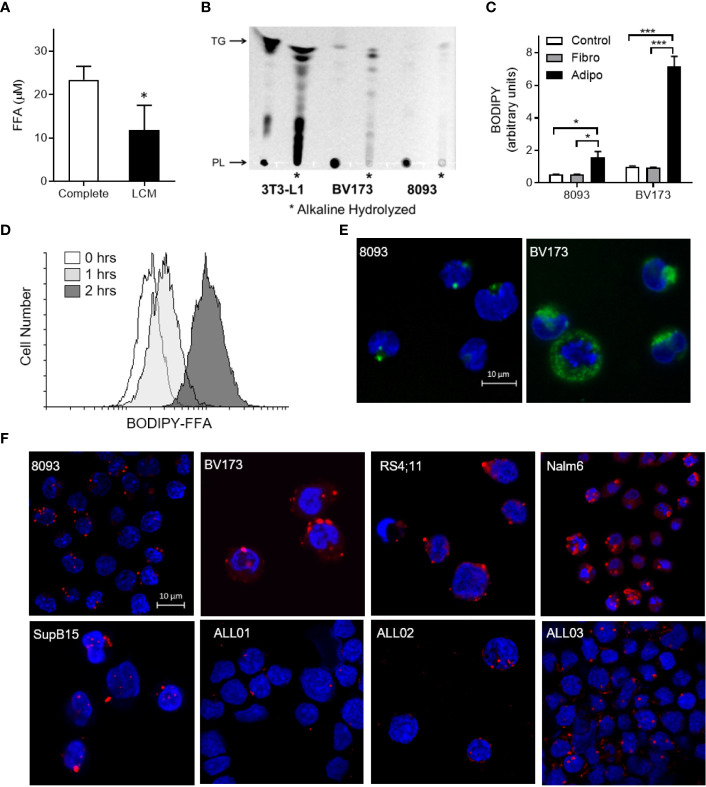
ALL cell uptake of adipocyte-derived FFA. **(A)** FFA concentration in complete media and BV173 LCM. (n=3; *p < 0.05). **(B)** Ultraviolet exposure of thin-layer chromatography plate with BODIPY-labeled lipids in 3T3-L1 adipocyte, BV173 and 8093 cells.*, Alkaline hydrolyzed lipid extracts from each cell; PL, Phospholipid; TG, Triglyceride. **(C)** BODIPY quantification in 8093 and BV173 ALL cells cultured for 48 hours in transwells alone (Control) or over fibroblasts (Fibro) or adipocytes (Adipo) pre-labeled with BODIPY-FFA. The median value of BODIPY fluorescence was determined by flow cytometry. (n=4/condition; *p < 0.05, ***p < 0.001) **(D)** Flow cytometry histogram of BV173 cells co-cultured for 0, 1 or 2 hours over BODIPY-FFA pre-labeled adipocytes. **(E)** Representative confocal images of ALL cells co-cultured for 48 hours over adipocytes pre-labeled with BODIPY-FFA. (Green: BODIPY-FFA; Blue: DAPI) **(F)** Oil Red O and DAPI stained murine and human ALL cell lines cultured under standard conditions, as well as leukemic blasts from patient-derived bone marrow smears (ALL01, ALL02, ALL03).

FFA concentrations were lower in media after culturing with ALL cells (LCM), indicating that ALL cells might take up exogenous FFA (**Figure 3A**). To test whether ALL cells take up adipocyte-derived FFA, we differentiated 3T3-L1 adipocytes in the presence of the fluorescent-labeled palmitate analogue, BODIPY-FFA. BODIPY fluorescence incorporated into adipocyte triglycerides and phospholipids (**Figure 3B**, 3T3-L1). ALL cells co-cultured over these labeled adipocytes accumulated BODIPY fluorescence (**Figures 3B, C**). Thin-layer chromatography demonstrated that fluorescence accumulated in ALL cell triglycerides (top band) and phospholipids (bottom band, mobilized by alkaline hydrolysis, **Figure 3B**). Fluorescent FFA from adipocytes appeared in ALL cells within one hour of co-culture (**Figure 3D**) and were visible in ALL cell membranes and cytoplasmic droplets (**Figure 3E**). Surprisingly, we found that ALL cells generally contain lipid droplets, as they were present in all five ALL cell lines tested and all three ALL patient bone marrow aspirate smears (**Figure 3F**).

### Adipocytes Preferentially Provide Unsaturated FFA to ALL Cells

**Figure 4:**
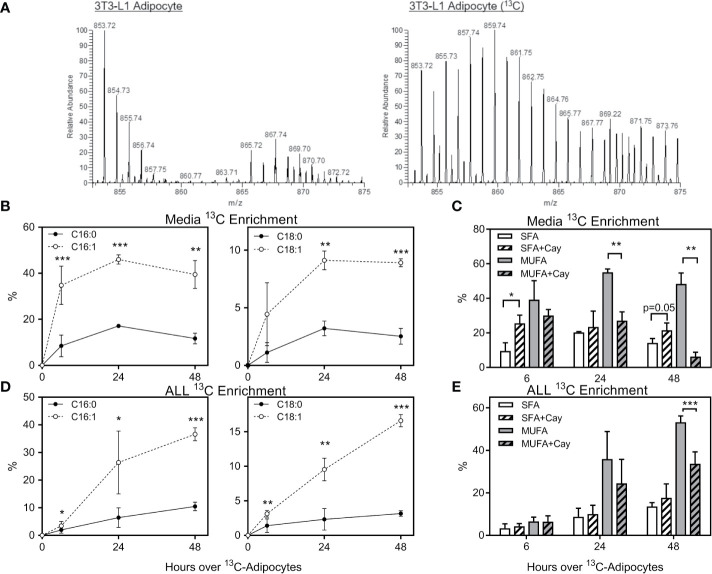
Stable-isotope lipidomic identification of adipocyte-derived FFA. **(A)** Representative spectra from nanoDESI-MS analysis of adipocytes differentiated without (left) and with (right) U-^13^C-glucose. Spectra are cropped to highlight triglycerides in the *m/z* range 850-875, and demonstrate the prevalence of ^13^C-enriched moieties. **(B)** Percent ^13^C_2_ enrichment of four FFA in media following adipocyte and ALL cell co-culture for 0, 6, 24 or 48 hours. Values are derived from average peak intensity for each FFA over 4 samplings. **(C)** ^13^C enrichment of media FFA following co-culture with adipocytes differentiated with U-^13^C-glucose in the presence or absence of the SCD1 inhibitor, Cay10566. Saturated FFA (SFA) represent the summation of C16:0 and C18:0, while monounsaturated FFA (MUFA) are the summation of C16:1 and C18:1 (n=4). *p < 0.05, **p < 0.01, ***p < 0.001. **(D, E)** ^13^C_2_ enrichment in FFA in BV173 ALL cells following adipocyte and ALL cell co-culture, as described for panels **(B, C)** above. *p < 0.05, **p < 0.01, ***p < 0.001.

To determine which FFA were being transferred from adipocytes to ALL cells, we differentiated 3T3-L1 adipocytes in U-^13^C-glucose, and then co-cultured these adipocytes under transwells containing 8093 ALL cells. We used nanoDESI-MS to quantify ^13^C incorporation into lipids as two-carbon units; high resolution MS provided sufficient resolving power to distinguish the small differences between ^13^C_2_-oleic acid and unlabeled stearic acid (**Figure S1**). Adipocytes showed a clear incorporation of label in their triglycerides (**Figure 4A**). In transwell co-cultures, ^13^C enrichment of C16 and C18 FFA in media steadily increased over the initial 24 hours before plateauing (**Figure 4B**). Interestingly, media ^13^C enrichment was significantly higher in unsaturated than in saturated FFA. Differentiating adipocytes in the presence of Cay10566, an inhibitor of the FFA desaturating enzyme, SCD1, led to less label in monounsaturated FFA and more in saturated FFA (**Figure 4C**). ALL cells co-cultured over ^13^C-labeled adipocytes showed ^13^C accumulation and enrichment in saturated and unsaturated FFA (**Figure 4D**), mirroring the enrichment observed in co-culture media (**Figure 4B**). Differentiating adipocytes with the SCD1 inhibitor partially blocked their preferential transfer of unsaturated FFA to ALL cells (**Figure 4E**).

### Adipocytes Relieve ALL Cell Dependence on De Novo Lipogenesis

**Figure 5:**
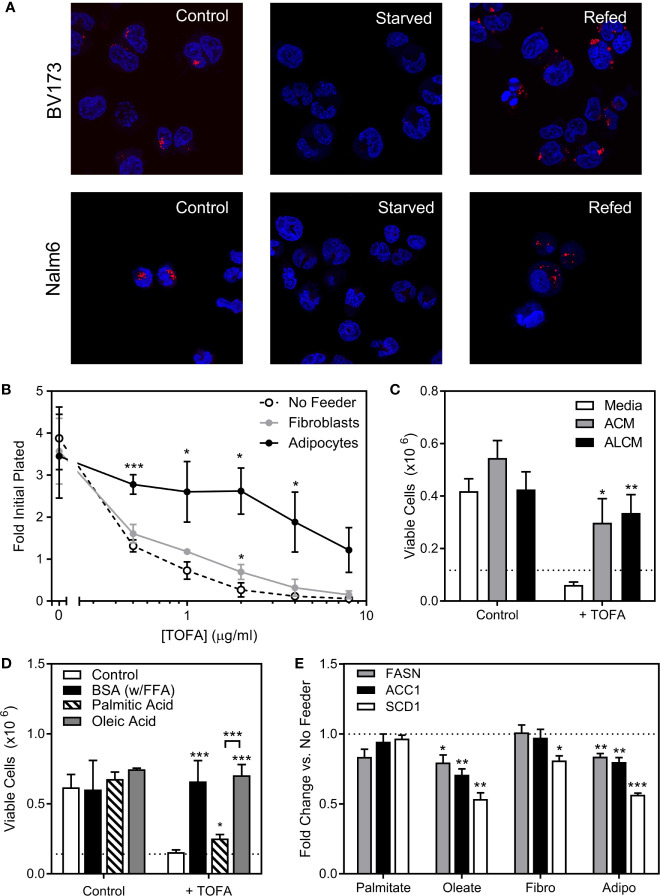
ALL cell dependence on *de novo* lipogenesis is relieved by adipocytes. **(A)** Confocal microscopy of BV173 and Nalm6 human ALL cells stained with Oil Red O after standard culture (Control), 24 hours in low-glucose serum-free medium (Starved), and 24 hours “refeeding” in standard medium after starvation (Refed). Representative of 6 pictures for each condition. **(B)** Viable BV173 cells after 72 hour co-culture in serum-free complete media with various doses of the ACC1 inhibitor, TOFA. (*p < 0.05, ***p < 0.001; n=3). **(C)** Viable BV173 cells after 72 hour culture in regular or conditioned media in the presence of 2 μg/mL TOFA (ACM, adipocyte-conditioned media; ALCM, adipocyte and leukemia cell-conditioned media; *p < 0.05, **p < 0.01 *vs.* media; n=3). **(D)** Viable BV173 cells after 72 hour culture in serum-free complete media supplemented with 1% BSA or 200 μM BSA-conjugated FFA, with and without 2 μg/mL TOFA (*p < 0.05, ***p < 0.001 *vs.* control; n=3). **(E)** Gene expression by rtPCR of *FASN*, *ACC1* and *SCD1* of BV173 ALL cells following 24 hour culture in serum free, low glucose media supplemented with 200μM exogenous FFA, or in co-culture with fibroblasts (Fibro) or adipocytes (Adipo) for 72 hours. Genes are normalized to β-actin before normalization to media only condition (*p < 0.05, **p < 0.01, ***p < 0.001, n=4).

We next investigated the role of endogenous sources in ALL cell lipid metabolism. ALL cell lipid droplets largely disappeared from BV173 and Nalm6 ALL cells after 24-hour culture in low glucose, serum-free media, and returned after 24 hours of culture back in standard media (**Figure 5A**), implying that ALL utilizes lipid droplets in times of metabolic need. We next inhibited the lipid synthetic enzymes ACC1 and SCD1 in ALL cells using TOFA (5-(Tetradecyloxy)-2-furoic acid) (18). In lipid-depleted media, TOFA was cytotoxic to BV173 cells, while this was reversed in the presence of adipocytes (**Figure 5B**), adipocyte-conditioned media (ACM, **Figure 5C**), or FFA-containing BSA (**Figure 5D**). While oleic acid completely reversed TOFA cytotoxicity, saturated palmitic acid offered only minimal protection (**Figure 5D**), confirming the importance of unsaturated FFA to ALL cell survival. In low glucose, serum-free media, gene expression of the key lipogenesis enzymes *FASN*, *ACC1* and *SCD1* were reduced in the presence of oleic acid and by co-culture over adipocytes, but were unaffected by exogenous palmitic acid (**Figure 5E**). Together, these data show that adipocytes can relieve ALL cell dependence on endogenous lipogenesis.

### ALL Cells Utilize Adipocyte-Derived FFA for Oxidative Phosphorylation

**Figure 6:**
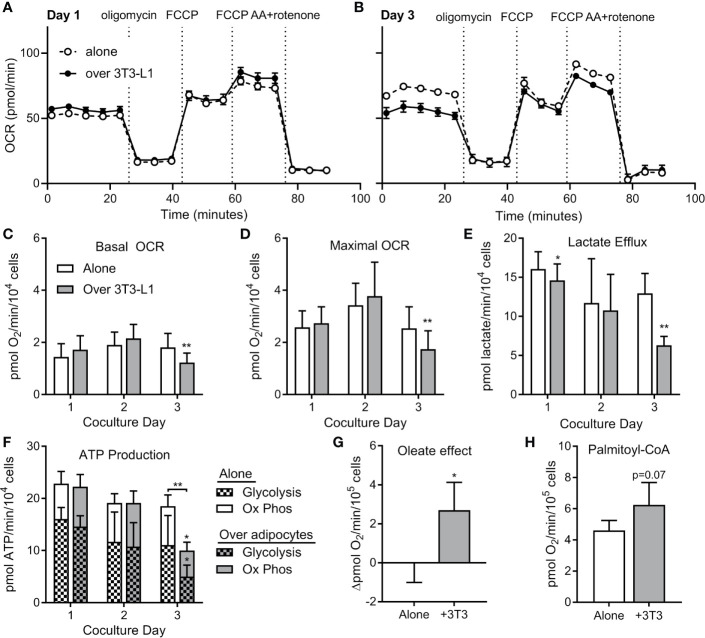
ALL cells rely on FFA oxidation in the presence of adipocytes. **(A)** Representative tracing of BV173 ALL cell O_2_ consumption rates (OCR) measured during extracellular flux analysis using the Seahorse XF96 analyzer. ALL cells had been cultured for one day in transwells alone or over 3T3-L1 adipocytes until just prior to analysis (n=4-6). **(B)** Representative day #3 tracing from experiments similar to A above (n=4-6) **(C, D)** ATP-linked basal **(C)** and maximal **(D)** OCR from experiments described above. *’s indicate significance *vs.* alone condition. **(E)** Lactate efflux was calculated from extracellular acidification rate (ECAR) from above experiments. **(F)** ATP production rates in ALL cells from experiments above. On day 3, glycolysis, oxidative phosphorylation, and total ATP production were lower in ALL cells cultured over adipocytes. **(G)** Difference in basal oxygen consumption of BV173 cells measured without and with BSA-conjugated oleic acid, after culture alone or over adipocytes (n=3). **(H)** Oxygen consumption rates for permeabilized BV173 ALL cells offered palmitoyl-CoA after culture alone or over adipocytes (n=3). *p < 0.05, **p < 0.01.

To test whether ALL cell metabolism is altered by nearby adipocytes, we measured rates of respiration and lactate efflux using a Seahorse XF analyzer. BV173 ALL cells were cultured in transwells over 3T3-L1 adipocytes, then transferred and acutely adhered to XF plates for standard bioenergetic analysis in experimental medium containing glucose, glutamine and pyruvate. Mitochondrial bioenergetics were largely unchanged after 24 or 48 hours of co-culture, as rates of basal and maximal uncoupler-stimulated respiration were not altered. However, co-culture with adipocytes over 72 hours caused a significant decrease in these respiratory parameters, indicating a reduced ability of these fuels to drive mitochondrial energetics (**Figures 6A–D**). Lactate efflux was also lower in ALL cells cultured over adipocytes (**Figure 6E**), demonstrating a reduced rate of glycolysis and further highlighting the reduced capacity for glucose oxidation in ALL cells after adipocyte co-culture. Consistent with these findings, ALL cells co-cultured over adipocytes had lower total ATP production rates, as well as ATP production rates from glycolysis and oxidative phosphorylation (**Figure 6F**).

We then hypothesized that the reduction in bioenergetics in standard assay conditions observed upon adipocyte co-culture was due to a switch in substrate preference towards fatty acids, which were not present in the experimental medium. Therefore, we offered ALL cells BSA-conjugated oleate as fuel to determine whether preference for fatty acids was increased upon 72 hour co-culture of ALL cells with differentiated adipocytes. Indeed, we observed a significant increase in oleate-driven basal respiration in co-cultured ALL cells over-and-above BSA controls that was entirely absent in ALL cells cultured alone (**Figure 6G**). To directly assess the capacity of *in situ* mitochondria to oxidize long chain fatty acids, we measured respiration in permeabilized ALL cells offered palmitoyl-CoA and carnitine. Indeed, permeabilized ALL cells that had been cultured with adipocytes trended toward a higher respiration rate than those cultured alone (**Figure 6H**). Thus, coculture with adipocytes causes ALL cells to decrease their capacity for oxidative metabolism of glucose and glutamine, but increase their capacity for FFA oxidation. Taken together, coculture with adipocytes causes a bioenergetic switch in ALL cells shifting substrate preference away from oxidation of glucose/glutamine and towards FFA oxidation.

**Figure 7:**
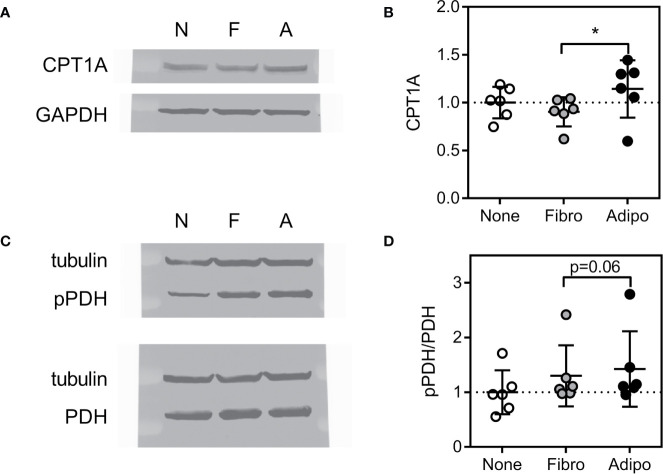
Expression of proteins regulating lipid metabolism in ALL cells **(A)** Representative western blot of BV173 lysate following culture in serum-free media alone or with fibroblasts or adipocytes for 72 hours. **(B)** Average CPT1A protein expression of BV173 cells described in A (*p<0.05, n=6). **(C)** Representative western blot of BV173 after culture as described above showing phospho- and total PDH. **(D)** Ratio of phospho-PDH to total PDH by densitometric analysis of western blots as in D (n=6).

Consistent with these findings, ALL cells cultured over adipocytes had higher expression of carnitine O-palmitoyl transferase 1A (CPT1A), largely considered the rate-controlling step in FFA oxidation (**Figures 7A, B**). Adipocyte co-culture was also associated with a tendency for increased ALL cell pyruvate dehydrogenase (PDH) phosphorylation (**Figures 7C, D**), signaling inhibition of this enzyme which catalyzes pyruvate entry into the Krebs cycle (38). Thus, adipocytes appear to shift metabolism of nearby ALL cells from glucose metabolism toward fatty acid oxidation.

### Oleic Acid Causes Modest Chemotherapy Resistance

**Figure 8:**
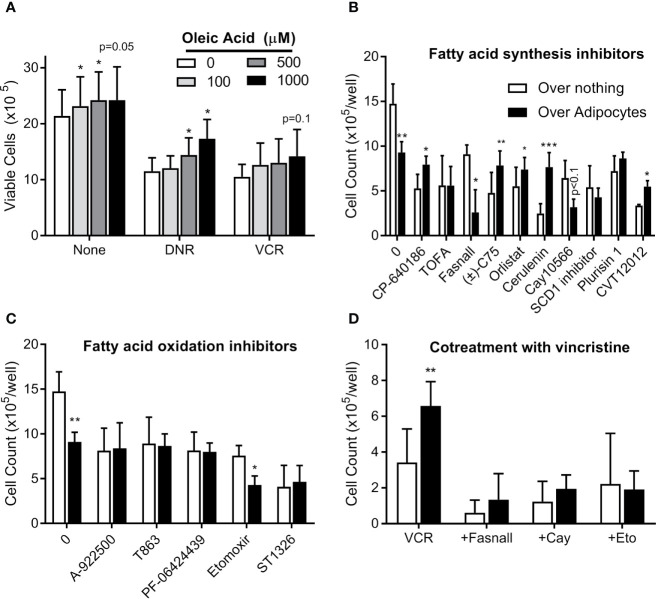
Targeting adipocyte protection of ALL cells **(A)** BV173 cells were cultured in serum free RPMI with 1% free fatty acid-free BSA, supplemented with various concentrations of oleic acid and treated with DNR or VCR for 72 hours. Low dose: DNR 25 nM, VCR 2 nM; high dose (not shown): DNR 60 nM, VCR 5 nM. **(B)** BV173 ALL cells were cultured in RPMI with 10% FBS in transwells over no feeder vs. 3T3-L1 adipocytes, and treated for 72 hours with inhibitors of fatty acid synthesis or desaturation, used at their estimated EC_50_ concentration. **(C)** BV173 ALL cells were treated as above, with inhibitors of fatty acid oxidation or triglyceride synthesis at their EC_50_ concentration. **(D)** BV173 ALL cells were treated with 0.7 nM vincristine alone or in combination with selected lipid-targeting drugs in transwells alone or over 3T3-L1 adipocytes. *p < 0.05, **p < 0.01, ***p < 0.001; n=4-5 for all experiments.

We next tested whether exogenous FFA could cause ALL cell chemotherapy resistance. In serum free media, oleic acid slightly increased BV173 cell proliferation and caused modest resistance to low (**Figure 8A**), but not high dose (not shown) daunorubicin and vincristine. Surviving cells increased with increasing concentrations of oleic acid. Thus, exogenous FFA could potentially contribute to modest chemotherapy resistance in microenvironments where levels are suboptimal.

### Targeting Lipid Metabolism Can Reverse Adipocyte Protection of ALL

We next screened 18 drugs targeting various aspects of lipid metabolism, including FFA uptake, synthesis, desaturation, incorporation into triglycerides, and oxidative metabolism. Dose responses were performed for each drug on BV173 ALL cells in monoculture (**Figure S2**). Drugs were then tested at their ~EC_50_ on ALL cells cultured in transwells alone or over 3T3-L1 adipocytes. Adipocytes protected ALL cells from five of the ten drugs targeting FFA synthesis, including inhibitors of ACC (CP-640186), FASN (C75, orlistat, cerulenin), and SCD1 (CVT12012, **Figure 8B**). This is consistent with the hypothesis that adipocyte FFA relieve ALL cells from their dependence on endogenous lipid synthesis. In contrast, adipocytes did not protect ALL from drugs targeting triglyceride synthesis or FFA oxidation (**Figure 8C**). Of interest, adipocytes actually increased ALL cell killing by three drugs: fasnall (FASN inhibitor), CAY10566 (SCD1 inhibitor; p=0.075), and etomoxir (CPT1 inhibitor). Further, these three drugs reversed adipocyte protection of ALL cells from vincristine (**Figure 8D**).

## Discussion

In the present study, we elucidate a previously unknown relationship between ALL and adipocytes, where ALL cells induce adipocyte release of FFA, which are taken up and used by ALL cells to incorporate into phospholipids, store in lipid droplets, relieve endogenous lipogenesis, and provide energy through beta-oxidation. ALL cells depend particularly on unsaturated FFA, and exogenous oleic acid contributes to a modest chemotherapy resistance against vincristine and daunorubicin. In this way, adipocytes can promote environmentally-mediated chemotherapy resistance. Thus, adipocyte provision of FFA might contribute to the worse outcomes experienced by obese patients with ALL (4) and other malignancies (39, 40).

These studies add a new facet to the already complex relationship between ALL cells and adipocytes. Adipocytes attract ALL cells to migrate into adipose tissue *via* the chemokine CXCL-12 (13). There, they are relatively protected from chemotherapy due to survival signals (6), adipocyte provision of glutamine and asparagine (41), and adipose tissue accumulation (12, 42) and metabolism (12) of chemotherapies. Adipocyte provision of FFA in this protective microenvironment could also support ALL cell robustness and chemotherapy resistance. Obese patients with excess adiposity have more potential microenvironments where ALL cells can engraft, proliferate, and resist chemotherapies. Further, obese individuals have more adipocytes in their bone marrow (43), the primary protective niche for ALL. Thus, these findings may help explain why obese ALL patients have worse outcomes (3, 5, 44).

We were unable to identify the signal(s) by which ALL cells induce lipolysis in nearby adipocytes. While we tested six candidate ALL-secreted cytokines, we were unable to recapitulate the effects of ALL conditioned media. It is possible that other cytokines we did not test are responsible; for example, adrenomedullin from breast cancer mammospheres has been shown to induce a CAA phenotype in adipocytes (45). Other ALL-secreted factors, such as metabolites or exosomes could also be responsible. Alternatively, ALL cell removal of metabolic fuels from the media could be a signal for adipocyte lipolysis. More work is needed to fully tease apart these two-way interactions between ALL and adipocytes.

Cancer cells proliferate rapidly, and thus have high lipid requirements for membrane synthesis and metabolism. Lipogenic enzymes like FASN and ACC1 are commonly overexpressed in cancer and associated with worse outcomes (46, 47). While ALL lipid requirements can be met by *de novo* lipogenesis, exogenous adipocyte-derived FFA can reduce reliance on this metabolically expensive process (**Figure 5**), and shift ALL cell fuel preference from glucose and more towards FFA metabolism (**Figures 6** and **7**). Further, we found that adipocytes preferentially transfer unsaturated FFA to ALL cells. Multiple studies have illustrated the importance of unsaturated FFA to cancer proliferation and survival, as well as the contrasting pro-apoptotic effects of saturated FFA (48–50). Indeed, the FFA desaturating enzyme, SCD1, is associated with cancer aggressiveness and poorer prognosis (51), and targeting this enzyme has shown promise against several cancers (16–18). Our study demonstrates that the ALL gene expression of lipogenesis enzymes FASN, ACC1, and SCD1 are reduced in the presence of adipocytes, reflecting a relative independence from endogenous lipogenesis and FFA desaturation.

Even without obesity, children with ALL have metabolic derangements that increase lipid availability. In one cohort, 99% of children with newly diagnosed ALL had dyslipidemia, with most exhibiting hypertriglyceridemia, irrespective of BMI (52). Current ALL chemotherapy regimens, specifically those containing L-asparaginase, are known to cause hypertriglyceridemia and hypercholesterolemia (53, 54). These transient increases in lipids could further facilitate ALL cell utilization of fatty acids.

Our findings are consistent with studies showing that cancer cells induce a phenotypic change in nearby adipocytes. Cancer associated adipocytes (CAA) surrounding solid tumors contain less lipid, express more inflammatory markers, and release cytokines that stimulate tumor cell adhesion, migration, and invasion (55–57). Cancer cells induce CAAs to release their lipids which are used by the tumor (55), similar to what we presently observe with ALL cells. Indeed, induction of adipocyte FFA release has been shown with acute myelogenous leukemia (11), ovarian cancer (10) and pancreatic cancer (58). Since adipocytes from obese individuals express higher levels of inflammatory cytokines, such as IL6 and TNFα (59), it is possible that obese adipocytes are already more “CAA-like” and more supportive of cancer cells, even before complete trans-differentiation into a distinct CAA phenotype.

These results reveal potential strategies that could be directed against environmentally-mediated chemotherapy resistance in ALL. Inhibition of FFA utilization has shown promise against a variety of cancers (16–22). In particular, targeting of FFA oxidation could reverse the advantage that cancer cells have in lipid rich environments. CPT1A inhibitors, such as etomoxir and ST1326, are effective against hematologic malignancies (19–22), and in our hands, nearby adipocytes actually enhanced the cytotoxicity of etomoxir against ALL (**Figure 8**). Conversely, the cytotoxicity of half of the fatty acid synthesis inhibitors we tested were reversed by adipocytes. Another strategy to consider is targeting of adipocyte lipolysis, perhaps by reducing inflammation within the tumor microenvironment or enhancing insulin sensitivity. TNFα inhibition can suppress breast cancer growth and metastasis (60, 61); whether this strategy acts directly on the cancer cells or indirectly on nearby cells in the local microenvironment is not completely clear. More work needs to be done to understand how these strategies are affected by adipocyte-rich microenvironments, particularly *in vivo*.

A weakness in the present study is that our human models of adipocytes did not demonstrate a robust ALL-cell induced lipolysis. This may be due to differences in the experimental models; in our hands, adipocytes differentiated from human SVF accumulate substantially less lipid than 3T3-L1 cells, making them less reflective of primary adipocytes *in vivo*. Primary adipocytes float, making them difficult to culture in standard conditions. Alternatively, it is possible that ALL cells do not induce as much lipolysis in human adipocytes as mouse. The difficulty of studying the ALL microenvironment *in situ* in humans makes teasing apart these possibilities challenging, and will likely require improved adipocyte and adipose tissue culturing techniques. Another related weakness is that our mechanistic findings have not yet been evaluated *in vivo*. Thus, more work is needed to confirm the human relevance of these findings.

In summary, we report that ALL cells stimulate FFA release from nearby adipocytes. Adipocyte FFA, particularly unsaturated FFA, are taken up by the ALL cells and incorporated into cell membranes and lipid droplets, used as a metabolic fuel, and contribute to modest chemotherapy resistance. Adipocyte FFA provision might particularly impair strategies that target endogenous lipid synthesis in cancer cells.

## Data Availability Statement

The raw data supporting the conclusions of this article will be made available by the authors, without undue reservation.

## Ethics Statement

The studies involving human participants were reviewed and approved by Children’s Hospital Los Angeles IRB and the UCLA IRB. Written informed consent to participate in this study was provided by the participants’ legal guardian/next of kin. The animal study was reviewed and approved by Children’s Hospital Los Angeles IACUC.

## Author Contributions

JT, TC, KM, RP, MC, RW, AJ, AD, C-CH, SN, and XS performed experiments and collected and analyzed data. JT, TC, AJ, AD, C-CH, KM, RZ, and SM made substantial contributions to the conception and design of the work. JT, TC, KM, MO, EO, RZ, and SM were involved in interpretation of the data. EO designed and oversaw all human subjects work. JT, TC, and SM drafted the work. All authors contributed to the article and approved the submitted version.

## Funding

This work was supported by the National Cancer Institute at the National Institutes of Health (R01 CA201444), The Saban Research Institute, The Children’s Leukemia Research Association, Inc, a Translational Research Program Award from the Leukemia & Lymphoma Society, and a generous gift from the Estate of Helen Dyess. ASD is supported by the National Institutes of Health (R35 GM138003). AJ is supported by the UCLA Tumor Cell Biology Training Program (T32CA009056). None of these funding bodies played a role in the study design, the collection, analysis, or interpretation of data, or the writing of the manuscript.

## Conflict of Interest

The authors declare that the research was conducted in the absence of any commercial or financial relationships that could be construed as a potential conflict of interest.

## Supplementary Material

Supplementary Figure 1 FFA resolving ability of nanoDESI-MS. Representative spectra from nanoDESI-MS analysis of ALL cells following co-culture with adipocytes differentiated without (left) and with (right) U-^13^C-glucose. Magnification of spectra at *m/z* 278-285 are shown to demonstrate the efficacy of LTQ-Orbitrap-XL mass spectrometer in resolving unenriched stearic acid from ^13^C_2_-substituted oleic acid, as separated by 0.0085 *m/z*.

Supplementary Figure 2 Inhibition of lipid metabolism in ALL cells. **(A)** Scheme showing major enzymes involved in FFA uptake and metabolism in ALL cells. ACC, Acetyl-Coa Carboxylase; ATGL, Adipose triglyceride lipase; DGAT, Diacylglycerol acyltransferase; FASN, Fatty acid synthase; HSL, Hormone-sensitive lipase; SCD, Stearoyl-CoA Desaturase. **(B–T)** Dose responses and calculated IC_50_ of drugs targeting lipid metabolism on BV173 ALL cells.

## Abbreviations

ACC, acetyl-CoA carboxylase; ALL, acute lymphoblastic leukemia; CCL, C-C motif chemokine ligand; FFA, free fatty acid; IL, interleukin; PDGF, platelet-derived growth factor AA; PDH, pyruvate dehydrogenase; SCD-1, steroyl-CoA decarboxylase-1; TNFα, tumor necrosis factor α; TOFA, (5-(tetradecyloxy)-2-furoic acid).
