# Supplementary figures and images for: Adipocytes Provide Fatty Acids to Acute Lymphoblastic Leukemia Cells
Source: Front Oncol. 2021 Apr 22;11:665763. doi: 10.3389/fonc.2021.665763 (PMC8100891; doi:10.3389/fonc.2021.665763)

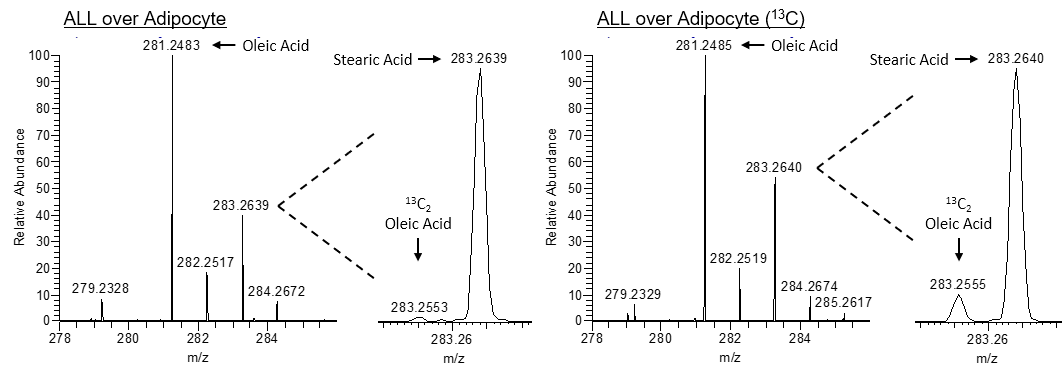

Supplement: Supplementary Figure 1 — FFA resolving ability of nanoDESI-MS. Representative spectra from nanoDESI-MS analysis of ALL cells following co-culture with adipocytes differentiated without (left) and with (right) U-13C-glucose. Magnification of spectra at m/z 278-285 are shown to demonstrate the efficacy of LTQ-Orbitrap-XL mass spectrometer in resolving unenriched stearic acid from 13C2-substituted oleic acid, as separated by 0.0085 m/z. [file Image_1.tif]

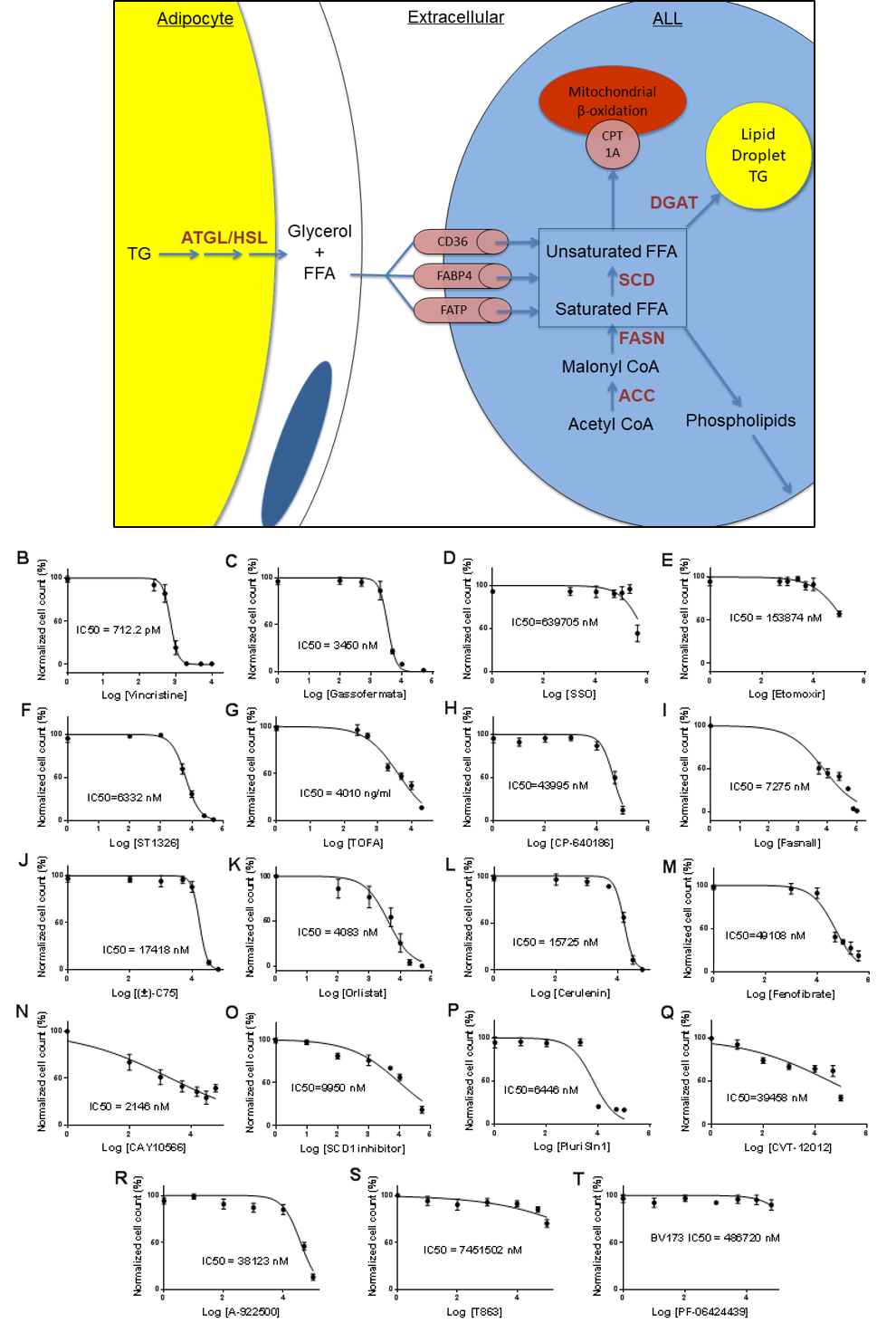

Supplement: Supplementary Figure 2 — Inhibition of lipid metabolism in ALL cells. (A) Scheme showing major enzymes involved in FFA uptake and metabolism in ALL cells. ACC, Acetyl-Coa Carboxylase; ATGL, Adipose triglyceride lipase; DGAT, Diacylglycerol acyltransferase; FASN, Fatty acid synthase; HSL, Hormone-sensitive lipase; SCD, Stearoyl-CoA Desaturase. (B–T) Dose responses and calculated IC50 of drugs targeting lipid metabolism on BV173 ALL cells. [file Image_2.tif]
