# Supplementary material for: Adipocytes Provide Fatty Acids to Acute Lymphoblastic Leukemia Cells
Source: Front Oncol. 2021 Apr 22;11:665763. doi: 10.3389/fonc.2021.665763 (PMC8100891; doi:10.3389/fonc.2021.665763)
Supplement: Supplementary file 3 [file Table_1.docx]

| Supplemental Table: Concentration of cytokines in media vs. LCM (pg/mL) | | | |
| --- | --- | --- | --- |
| **Cytokine** | **RPMI (10% FBS)** | **LCM** | **p value** |
| Fractalkine | 6.68 ± 3.88 | 19.82 ± 3.36 | 0.009 |
| I-309 | 0.16 ± 0.00 | 0.30 ± 0.04 | 0.002 |
| IFNα2 | 1.34 ± 0.85 | 2.96 ± 0.78 | 0.055 |
| IL-1β | 0.08 ± 0.07 | 0.28 ± 0.11 | 0.017 |
| IL-4 | 0.59 ± 0.74 | 1.99 ± 1.09 | 0.076 |
| IL-6^1^ | 0.10 ± 0.01 | 0.13 ± 0.02 | 0.040 |
| IL-9 | 0.08 ± 0.02 | 0.19 ± 0.04 | 0.001 |
| IL-10 | 0.31 ± 0.03 | 0.44 ± 0.13 | 0.099 |
| IL-16 | 4.50 ± 0.18 | 9.94 ± 2.68 | 0.010 |
| IL-18 | 0 | 0.81 ± 0.38 | 0.009 |
| MCP-1 | 1.07 ± 0.72 | 67.7 ± 15.1 | 0.001 |
| MCP-4 | 4.03 ± 0.00 | 5.59 ± 1.22 | 0.045 |
| MIP-1α | 0 | 4.01 ± 0.90 | 0.001 |
| PDGF-AA^2^ | 0.09 ± 0.01 | 2277 ± 1649 | 0.037 |
| PDGF-BB | 0.14 ± 0.22 | 0.96 ± 0.13 | 0.011 |
| RANTES^1^ | 0 | 151.2 ± 70.2 | 0.009 |
| TARC | 0.07 ± 0.02 | 7.94 ± 6.74 | 0.059 |
| TNFα^1^ | 0.15 ± 0.02 | 5.66 ± 2.81 | 0.012 |
| TNFβ | 0.07 ± 0.10 | 1.20 ± 0.41 | 0.002 |
| VEGF-A | 0.87 ± 0.76 | 4.18 ± 2.49 | 0.039 |
| ^1^Measures below the lowest standard were substituted with zero; ^2^measures above the highest standard were substituted with the highest standard value. | | | |
